# 2-(4-Chloro­phen­yl)-1,5-diphenyl-3-tosyl­imidazolidin-4-one

**DOI:** 10.1107/S1600536811008427

**Published:** 2011-03-12

**Authors:** S. Ranjith, A. SubbiahPandi, K. Namitharan, K. Pitchumani

**Affiliations:** a Department of Physics, Presidency College (Autonomous), Chennai 600 005, India; b School of Chemistry, Madurai Kamaraj University, Madurai 625 021, India

## Abstract

In the title compound, C_28_H_23_ClN_2_O_3_S, the central imidazolidine ring adopts a twisted conformation and the S atom has distorted tetra­hedral geometry. The crystal packing is stabilized by C—H⋯O, C—H⋯π and π–π inter­actions [centroid–centroid distance = 3.8302 (10) Å].

## Related literature

For the biological activity of sulfonamides, see: Zareef *et al.* (2007 ▶); Chohan *et al.* (2007 ▶); Pomarnacka & Kozlarska-Kedra (2003 ▶); Nieto *et al.* (2005 ▶); Wang *et al.* (1995 ▶). For a related structure, see: Chakkaravarthi *et al.* (2008 ▶). For puckering parameters, see: Cremer & Pople (1975 ▶). For asymmetry parameters, see: Nardelli *et al.* (1983 ▶). For graph-set notation, see: Bernstein *et al.* (1995 ▶).
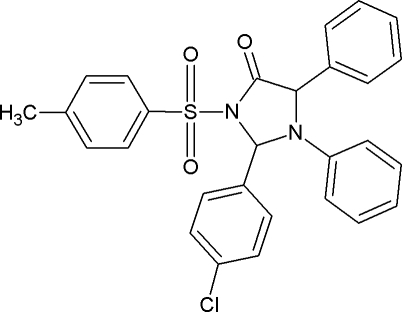


## Experimental

### 

#### Crystal data

C_28_H_23_ClN_2_O_3_S
*M* *_r_* = 503.00
Monoclinic, 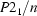

*a* = 10.8458 (3) Å
*b* = 13.0191 (4) Å
*c* = 17.6720 (5) Å
β = 103.757 (2)°
*V* = 2423.75 (12) Å^3^
*Z* = 4
Mo *K*α radiation
μ = 0.28 mm^−1^
*T* = 293 K
0.25 × 0.22 × 0.19 mm

#### Data collection

Bruker APEXII CCD area-detector diffractometer
Absorption correction: multi-scan (*SADABS*; Sheldrick, 1996 ▶) *T*_min_ = 0.981, *T*_max_ = 0.985
32315 measured reflections
7313 independent reflections
5098 reflections with *I* > 2σ(*I*)
*R*_int_ = 0.030

#### Refinement

*R*[*F* ^2^ > 2σ(*F* ^2^)] = 0.042
*wR*(*F* ^2^) = 0.115
*S* = 1.05
7313 reflections
317 parameters
H-atom parameters constrained
Δρ_max_ = 0.28 e Å^−3^
Δρ_min_ = −0.35 e Å^−3^

### 

Data collection: *APEX2* (Bruker, 2004 ▶); cell refinement: *SAINT* (Bruker, 2004 ▶); data reduction: *SAINT*; program(s) used to solve structure: *SHELXS97* (Sheldrick, 2008 ▶); program(s) used to refine structure: *SHELXL97* (Sheldrick, 2008 ▶); molecular graphics: *ORTEP-3* (Farrugia, 1997 ▶); software used to prepare material for publication: *SHELXL97* and *PLATON* (Spek, 2009 ▶).

## Supplementary Material

Crystal structure: contains datablocks global, I. DOI: 10.1107/S1600536811008427/pk2300sup1.cif

Structure factors: contains datablocks I. DOI: 10.1107/S1600536811008427/pk2300Isup2.hkl

Additional supplementary materials:  crystallographic information; 3D view; checkCIF report

## Figures and Tables

**Table 1:** Hydrogen-bond geometry (Å, °) *Cg*2 and *Cg*4 are the centroids of the C2–C7 and C15–C20 rings, respectively.

*D*—H⋯*A*	*D*—H	H⋯*A*	*D*⋯*A*	*D*—H⋯*A*
C8—H8⋯O3^i^	0.98	2.44	3.3659 (17)	158
C14—H14⋯O3^i^	0.93	2.57	3.3855 (19)	146
C24—H24⋯O2^ii^	0.93	2.59	3.289 (2)	132
C1—H1*C*⋯*Cg*4^iii^	0.96	2.90	3.484 (2)	120
C11—H11⋯*Cg*2^iii^	0.93	2.88	3.619 (17)	138

Symmetry codes: (i) 
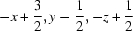
; (ii) 
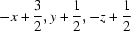
; (iii) 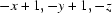.

**Fig. 1.**
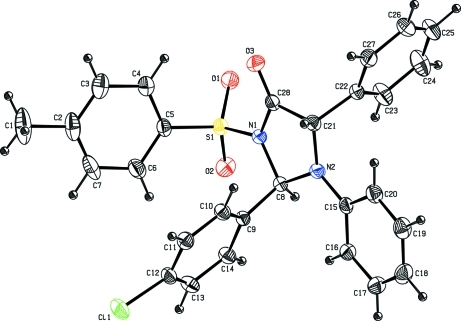
The structure of the title compound showing the atom-numbering scheme. The displacement ellipsoids are drawn at the 30% probability level.

**Fig. 2.**
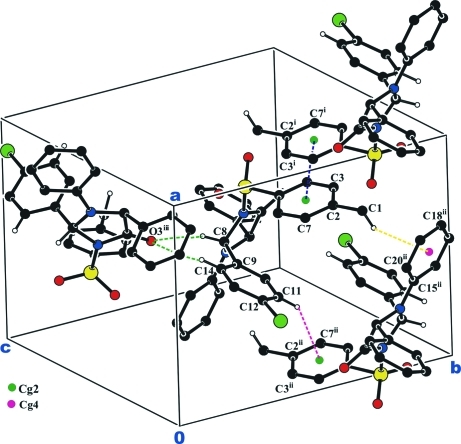
π—π, C–H···π interactions (dotted lines) in the title compound and also bifurcated hydrogen bonds formed by C8–H8···O3 and C14–H14···O3 at 3/2 - x,-1/2 + y,1/2 - z, results in the formation of R21(6) [O3,H8,C8,C9,C14,H14] bifurcated ring. Cg denotes ring centroid.[Symmetry code: (i) 2 - x,1 - y,-z;(ii) 1 - x,1 - y,-z; (iii) 3/2 - x,-1/2 + y,1/2 - z.]

## supplementary crystallographic information

### Comment

Sulfonamides have widely been recognized for their wide variety of pharmacological activities such as antibacterial, antitumor, anti-carbonic anhydrase, diuretic, hypoglycaemic, antithyroid and protease inhibitory activity. Sulfonamides have also been used clinically as antimalarial agents (Zareef *et al.*, 2007), particularly sulfadiazine and sulfadoxine. Due to their significant pharmacology applications and widespread use in medicine, these compounds have also gained attention in bioinorganic and metal-based (Chohan *et al.*, 2007) drug chemistry. Sulfonamide derivatives are well known drugs and are used to control diseases caused by bacterial infections. Benzene sulfonamide derivatives are known to exhibit anticancer and HIV activities (Pomarnacka & Kozlarska-Kedra, 2003) and antibacterial activities (Nieto *et al.*, 2005). Imidazolidine compounds are important intermediates and building blocks in the construction of various biologically active compounds (Wang *et al.*, 1995). Against this background, and in order to obtain detailed information on molecular conformations in the solid state, an X-ray study of the title compound was carried out.

X-ray analysis confirms the molecular structure and atom connectivity as illustrated in Fig. 1. The geometry around the S atom is distorted tetrahedral, comprising two O atoms of the sulfonyl group, a C atom of a benzene ring and the imidazolidine N atom. The S–O, S–C, and S–N distances are 1.418 (1), 1.747 (1) and 1.677 (1) Å, respectively. These are comparable to those in similar structures (Chakkaravarthi *et al.*, 2008). The atom Cl1 deviates by 0.130 (1)Å from the least-squares plane of the ring C9–C14. The S atom exhibits significant deviation from that of a regular tetrahedron, with the largest deviations for the O–S–O [O1–S1–O2 120.9 (7)°] and O–S–N angles [O1–S1–N1 106.5 (6)°]. The widening of the angles may be due to repulsive interactions between the two short S=O bonds, similar to what is observed in related structures (Chakkaravarthi *et al.*, 2008). The chlorobenzene ring makes the dihedral angles of 39.4 (8), 85.1 (8) and 1.9 (9)° with respect to the C2–C7, C15–C20 and C23–C28 benzene rings.

The imidazolidine ring adopts a twisted conformation, with puckering parameters q_2_ and φ (Cremer & Pople, 1975) and the smallest displacement asymmetric parameters, Δ, (Nardelli *et al.*, 1983) as follows: q_2_ = 0.1300 (14) Å, φ = 21.9 (6)°, Δ_s_(C8) = 4.70 (15). The intramolecular C6–H6···O2 hydrogen bond completes a five-membered ring, which generates an S(5) motif (Bernstein *et al.*, 1995). Atoms C8 and C14 act as donors to form bifurcated hydrogen bonds with atom O3 as an acceptor, results in the formation of *R*^2^_1_(6) bifurcated ring. In addition to van der Waals interactions, the crystal packing is stabilized by C–H..O, C–H···π and π···π interactions (Table. 1).

### Experimental

4-Toluenesulfonyl azide (1.3 mmol), phenylacetylene (1.2 mmol), 4-chlorophenyl *N*-phenylnitrone (1.0 mmol) and triethylamine (2 mmol) were successively added to Cu^1^—Y zeolite (30 mg) in dichloromethane under N_2_ atmosphere. After stirring at room temperature for 6 h, the mixture was diluted with dichloromethane. After removing the catalyst by filtration, followed by solvent evaporation, the resulting crude product was finally purified by column chromatography (silica gel). Single crystals suitable for X-ray diffraction were obtained by slow evaporation of a solution of the title compound in acetone at room temperature.

### Refinement

All H atoms were fixed geometrically and allowed to ride on their parent C atoms, with C—H distances fixed in the range 0.93–0.97 Å with *U*_iso_(H) = 1.5*U*_eq_(C) for methyl H 1.2*U*_eq_(C) for other H atoms.

### Crystal data

**Table:**

C_28_H_23_ClN_2_O_3_S	*F*(000) = 1048
*M**_r_* = 503.00	*D*_x_ = 1.378 Mg m^−^^3^
Monoclinic, *P*2_1_/*n*	Mo *K*α radiation, λ = 0.71073 Å
Hall symbol: -P 2yn	Cell parameters from 7313 reflections
*a* = 10.8458 (3) Å	θ = 2.4–30.4°
*b* = 13.0191 (4) Å	µ = 0.28 mm^−^^1^
*c* = 17.6720 (5) Å	*T* = 293 K
β = 103.757 (2)°	Block, colourless
*V* = 2423.75 (12) Å^3^	0.25 × 0.22 × 0.19 mm
*Z* = 4	

### Data collection

**Table:**

Bruker APEXII CCD area-detector diffractometer	7313 independent reflections
Radiation source: fine-focus sealed tube	5098 reflections with *I* > 2σ(*I*)
graphite	*R*_int_ = 0.030
ω and φ scans	θ_max_ = 30.4°, θ_min_ = 2.4°
Absorption correction: multi-scan (*SADABS*; Sheldrick, 1996)	*h* = −15→15
*T*_min_ = 0.981, *T*_max_ = 0.985	*k* = −17→18
32315 measured reflections	*l* = −22→25

### Refinement

**Table:**

Refinement on *F*^2^	Primary atom site location: structure-invariant direct methods
Least-squares matrix: full	Secondary atom site location: difference Fourier map
*R*[*F*^2^ > 2σ(*F*^2^)] = 0.042	Hydrogen site location: inferred from neighbouring sites
*wR*(*F*^2^) = 0.115	H-atom parameters constrained
*S* = 1.05	*w* = 1/[σ^2^(*F*_o_^2^) + (0.0495*P*)^2^ + 0.5408*P*] where *P* = (*F*_o_^2^ + 2*F*_c_^2^)/3
7313 reflections	(Δ/σ)_max_ = 0.001
317 parameters	Δρ_max_ = 0.28 e Å^−^^3^
0 restraints	Δρ_min_ = −0.35 e Å^−^^3^

### Special details

**Table:**

Geometry. All e.s.d.'s (except the e.s.d. in the dihedral angle between two l.s. planes) are estimated using the full covariance matrix. The cell e.s.d.'s are taken into account individually in the estimation of e.s.d.'s in distances, angles and torsion angles; correlations between e.s.d.'s in cell parameters are only used when they are defined by crystal symmetry. An approximate (isotropic) treatment of cell e.s.d.'s is used for estimating e.s.d.'s involving l.s. planes.
Refinement. Refinement of *F*^2^ against ALL reflections. The weighted *R*-factor *wR* and goodness of fit *S* are based on *F*^2^, conventional *R*-factors *R* are based on *F*, with *F* set to zero for negative *F*^2^. The threshold expression of *F*^2^ > σ(*F*^2^) is used only for calculating *R*-factors(gt) *etc*. and is not relevant to the choice of reflections for refinement. *R*-factors based on *F*^2^ are statistically about twice as large as those based on *F*, and *R*- factors based on ALL data will be even larger.

### Fractional atomic coordinates and isotropic or equivalent isotropic displacement parameters (Å^2^)

**Table:**

	*x*	*y*	*z*	*U*_iso_*/*U*_eq_	
C1	0.8204 (2)	0.6211 (2)	−0.10561 (13)	0.0944 (10)	
H1A	0.8293	0.5757	−0.1468	0.142*	
H1B	0.8843	0.6735	−0.0987	0.142*	
H1C	0.7378	0.6523	−0.1188	0.142*	
C2	0.83550 (16)	0.5610 (2)	−0.03116 (10)	0.0615 (6)	
C3	0.87311 (18)	0.60888 (16)	0.03995 (11)	0.0592 (5)	
H3	0.8903	0.6789	0.0417	0.071*	
C4	0.88600 (16)	0.55548 (13)	0.10884 (10)	0.0477 (4)	
H4	0.9107	0.5892	0.1565	0.057*	
C5	0.86169 (13)	0.45127 (12)	0.10598 (8)	0.0370 (3)	
C6	0.82418 (16)	0.40127 (16)	0.03542 (10)	0.0524 (4)	
H6	0.8079	0.3311	0.0334	0.063*	
C7	0.81134 (17)	0.4579 (2)	−0.03242 (10)	0.0649 (6)	
H7	0.7855	0.4248	−0.0802	0.078*	
C8	0.65428 (12)	0.31035 (10)	0.20980 (7)	0.0301 (3)	
H8	0.6929	0.2445	0.2291	0.036*	
C9	0.58939 (12)	0.30175 (10)	0.12399 (8)	0.0298 (3)	
C10	0.51246 (13)	0.37975 (11)	0.08615 (8)	0.0359 (3)	
H10	0.4942	0.4355	0.1145	0.043*	
C11	0.46242 (14)	0.37599 (12)	0.00672 (9)	0.0396 (3)	
H11	0.4102	0.4284	−0.0184	0.048*	
C12	0.49087 (14)	0.29372 (12)	−0.03472 (8)	0.0398 (3)	
C13	0.56268 (16)	0.21323 (13)	0.00174 (9)	0.0455 (4)	
H13	0.5782	0.1566	−0.0268	0.055*	
C14	0.61182 (14)	0.21732 (11)	0.08156 (9)	0.0389 (3)	
H14	0.6602	0.1629	0.1068	0.047*	
C15	0.46241 (13)	0.29610 (11)	0.26339 (8)	0.0336 (3)	
C16	0.43733 (14)	0.19730 (12)	0.23351 (9)	0.0388 (3)	
H16	0.4952	0.1644	0.2104	0.047*	
C17	0.32626 (16)	0.14799 (14)	0.23816 (10)	0.0491 (4)	
H17	0.3105	0.0818	0.2185	0.059*	
C18	0.23885 (16)	0.19555 (16)	0.27141 (11)	0.0551 (5)	
H18	0.1638	0.1623	0.2735	0.066*	
C19	0.26360 (15)	0.29236 (15)	0.30142 (10)	0.0516 (4)	
H19	0.2050	0.3246	0.3242	0.062*	
C20	0.37404 (14)	0.34271 (13)	0.29837 (9)	0.0429 (3)	
H20	0.3898	0.4081	0.3197	0.052*	
C21	0.60120 (14)	0.45092 (11)	0.28719 (8)	0.0353 (3)	
H21	0.5306	0.4961	0.2630	0.042*	
C23	0.63258 (14)	0.46406 (11)	0.37521 (8)	0.0364 (3)	
C28	0.70564 (19)	0.39176 (15)	0.42268 (10)	0.0578 (5)	
H28	0.7302	0.3321	0.4013	0.069*	
C27	0.7424 (2)	0.40811 (19)	0.50230 (11)	0.0731 (6)	
H27	0.7914	0.3593	0.5344	0.088*	
C26	0.7067 (2)	0.49587 (19)	0.53377 (11)	0.0694 (6)	
H26	0.7324	0.5070	0.5872	0.083*	
C25	0.6339 (2)	0.56698 (16)	0.48732 (11)	0.0628 (5)	
H25	0.6089	0.6261	0.5092	0.075*	
C24	0.59660 (18)	0.55168 (13)	0.40753 (10)	0.0491 (4)	
H24	0.5472	0.6007	0.3759	0.059*	
N1	0.75179 (11)	0.39178 (9)	0.22299 (6)	0.0318 (2)	
N2	0.57393 (11)	0.34697 (10)	0.25969 (7)	0.0368 (3)	
O1	0.98232 (10)	0.43180 (9)	0.25004 (6)	0.0459 (3)	
O2	0.89588 (10)	0.27584 (9)	0.17703 (7)	0.0479 (3)	
C22	0.71777 (14)	0.47825 (11)	0.25818 (8)	0.0350 (3)	
S1	0.88693 (3)	0.38196 (3)	0.19302 (2)	0.03485 (10)	
Cl1	0.43640 (5)	0.29404 (4)	−0.13549 (2)	0.06360 (15)	
O3	0.77172 (11)	0.55951 (8)	0.26593 (7)	0.0480 (3)	

### Atomic displacement parameters (Å^2^)

**Table:**

	*U*^11^	*U*^22^	*U*^33^	*U*^12^	*U*^13^	*U*^23^
C1	0.0684 (13)	0.165 (3)	0.0561 (13)	0.0392 (16)	0.0268 (11)	0.0549 (15)
C2	0.0390 (9)	0.1076 (17)	0.0414 (10)	0.0192 (10)	0.0163 (7)	0.0250 (10)
C3	0.0596 (11)	0.0658 (12)	0.0559 (11)	0.0111 (9)	0.0209 (9)	0.0236 (9)
C4	0.0540 (9)	0.0509 (9)	0.0390 (8)	−0.0006 (8)	0.0131 (7)	0.0051 (7)
C5	0.0329 (7)	0.0504 (9)	0.0292 (7)	−0.0002 (6)	0.0099 (5)	0.0016 (6)
C6	0.0496 (9)	0.0714 (12)	0.0382 (8)	−0.0063 (8)	0.0148 (7)	−0.0099 (8)
C7	0.0507 (10)	0.1166 (19)	0.0283 (8)	0.0025 (11)	0.0110 (7)	−0.0052 (10)
C8	0.0323 (6)	0.0316 (7)	0.0269 (6)	−0.0013 (5)	0.0078 (5)	−0.0011 (5)
C9	0.0304 (6)	0.0323 (7)	0.0273 (6)	−0.0012 (5)	0.0081 (5)	−0.0016 (5)
C10	0.0387 (7)	0.0351 (7)	0.0338 (7)	0.0041 (6)	0.0087 (6)	−0.0029 (6)
C11	0.0372 (7)	0.0431 (8)	0.0359 (7)	0.0017 (6)	0.0033 (6)	0.0054 (6)
C12	0.0383 (7)	0.0527 (9)	0.0267 (7)	−0.0074 (7)	0.0041 (6)	−0.0027 (6)
C13	0.0533 (9)	0.0458 (9)	0.0369 (8)	0.0005 (7)	0.0097 (7)	−0.0129 (7)
C14	0.0440 (8)	0.0370 (8)	0.0343 (7)	0.0054 (6)	0.0067 (6)	−0.0031 (6)
C15	0.0331 (7)	0.0423 (8)	0.0247 (6)	−0.0014 (6)	0.0057 (5)	0.0036 (5)
C16	0.0390 (7)	0.0424 (8)	0.0338 (7)	−0.0032 (6)	0.0067 (6)	0.0011 (6)
C17	0.0489 (9)	0.0514 (9)	0.0452 (9)	−0.0129 (8)	0.0076 (7)	0.0015 (7)
C18	0.0379 (8)	0.0745 (13)	0.0525 (10)	−0.0143 (8)	0.0098 (7)	0.0071 (9)
C19	0.0374 (8)	0.0731 (12)	0.0467 (9)	0.0013 (8)	0.0149 (7)	0.0025 (8)
C20	0.0408 (8)	0.0524 (9)	0.0372 (8)	0.0006 (7)	0.0123 (6)	−0.0024 (7)
C21	0.0404 (7)	0.0353 (7)	0.0313 (7)	0.0007 (6)	0.0106 (6)	−0.0028 (6)
C23	0.0420 (7)	0.0387 (7)	0.0314 (7)	−0.0048 (6)	0.0144 (6)	−0.0054 (6)
C28	0.0711 (12)	0.0630 (11)	0.0390 (9)	0.0181 (9)	0.0122 (8)	−0.0029 (8)
C27	0.0816 (14)	0.0961 (16)	0.0380 (10)	0.0180 (13)	0.0067 (10)	0.0061 (10)
C26	0.0871 (15)	0.0886 (15)	0.0351 (9)	−0.0171 (12)	0.0198 (10)	−0.0148 (10)
C25	0.0907 (14)	0.0578 (11)	0.0477 (10)	−0.0143 (11)	0.0320 (10)	−0.0208 (9)
C24	0.0671 (11)	0.0402 (8)	0.0462 (9)	−0.0046 (8)	0.0255 (8)	−0.0058 (7)
N1	0.0336 (6)	0.0333 (6)	0.0298 (6)	−0.0039 (5)	0.0101 (5)	−0.0039 (5)
N2	0.0402 (6)	0.0400 (6)	0.0341 (6)	−0.0072 (5)	0.0165 (5)	−0.0096 (5)
O1	0.0361 (5)	0.0592 (7)	0.0382 (6)	−0.0085 (5)	0.0003 (4)	0.0051 (5)
O2	0.0454 (6)	0.0413 (6)	0.0606 (7)	0.0060 (5)	0.0200 (6)	−0.0006 (5)
C22	0.0427 (7)	0.0357 (7)	0.0269 (6)	−0.0005 (6)	0.0088 (6)	−0.0004 (5)
S1	0.03133 (17)	0.0400 (2)	0.03336 (18)	0.00022 (14)	0.00796 (13)	0.00157 (14)
Cl1	0.0700 (3)	0.0859 (4)	0.02902 (19)	−0.0131 (3)	0.00031 (19)	−0.0035 (2)
O3	0.0615 (7)	0.0359 (6)	0.0511 (7)	−0.0115 (5)	0.0225 (6)	−0.0069 (5)

### Geometric parameters (Å, °)

**Table:**

C1—C2	1.506 (3)	C15—N2	1.3941 (18)
C1—H1A	0.9600	C15—C20	1.397 (2)
C1—H1B	0.9600	C16—C17	1.385 (2)
C1—H1C	0.9600	C16—H16	0.9300
C2—C7	1.366 (3)	C17—C18	1.376 (3)
C2—C3	1.375 (3)	C17—H17	0.9300
C3—C4	1.380 (2)	C18—C19	1.369 (3)
C3—H3	0.9300	C18—H18	0.9300
C4—C5	1.381 (2)	C19—C20	1.378 (2)
C4—H4	0.9300	C19—H19	0.9300
C5—C6	1.380 (2)	C20—H20	0.9300
C5—S1	1.7478 (15)	C21—N2	1.4446 (19)
C6—C7	1.386 (3)	C21—C22	1.5154 (19)
C6—H6	0.9300	C21—C23	1.5207 (19)
C7—H7	0.9300	C21—H21	0.9800
C8—N2	1.4592 (17)	C23—C24	1.373 (2)
C8—N1	1.4764 (17)	C23—C28	1.379 (2)
C8—C9	1.5165 (18)	C28—C27	1.385 (3)
C8—H8	0.9800	C28—H28	0.9300
C9—C10	1.3817 (19)	C27—C26	1.366 (3)
C9—C14	1.3844 (19)	C27—H27	0.9300
C10—C11	1.380 (2)	C26—C25	1.359 (3)
C10—H10	0.9300	C26—H26	0.9300
C11—C12	1.373 (2)	C25—C24	1.386 (2)
C11—H11	0.9300	C25—H25	0.9300
C12—C13	1.372 (2)	C24—H24	0.9300
C12—Cl1	1.7377 (15)	N1—C22	1.3782 (18)
C13—C14	1.385 (2)	N1—S1	1.6776 (11)
C13—H13	0.9300	O1—S1	1.4181 (11)
C14—H14	0.9300	O2—S1	1.4181 (12)
C15—C16	1.393 (2)	C22—O3	1.2008 (17)
			
C2—C1—H1A	109.5	C15—C16—H16	120.0
C2—C1—H1B	109.5	C18—C17—C16	120.95 (17)
H1A—C1—H1B	109.5	C18—C17—H17	119.5
C2—C1—H1C	109.5	C16—C17—H17	119.5
H1A—C1—H1C	109.5	C19—C18—C17	119.23 (16)
H1B—C1—H1C	109.5	C19—C18—H18	120.4
C7—C2—C3	118.31 (17)	C17—C18—H18	120.4
C7—C2—C1	121.0 (2)	C18—C19—C20	121.00 (16)
C3—C2—C1	120.7 (2)	C18—C19—H19	119.5
C2—C3—C4	121.58 (19)	C20—C19—H19	119.5
C2—C3—H3	119.2	C19—C20—C15	120.34 (16)
C4—C3—H3	119.2	C19—C20—H20	119.8
C3—C4—C5	118.97 (17)	C15—C20—H20	119.8
C3—C4—H4	120.5	N2—C21—C22	103.10 (11)
C5—C4—H4	120.5	N2—C21—C23	115.32 (12)
C6—C5—C4	120.63 (15)	C22—C21—C23	108.53 (11)
C6—C5—S1	120.18 (13)	N2—C21—H21	109.9
C4—C5—S1	119.11 (12)	C22—C21—H21	109.9
C5—C6—C7	118.55 (19)	C23—C21—H21	109.9
C5—C6—H6	120.7	C24—C23—C28	119.54 (15)
C7—C6—H6	120.7	C24—C23—C21	120.15 (14)
C2—C7—C6	121.95 (18)	C28—C23—C21	120.13 (13)
C2—C7—H7	119.0	C23—C28—C27	119.88 (17)
C6—C7—H7	119.0	C23—C28—H28	120.1
N2—C8—N1	100.27 (10)	C27—C28—H28	120.1
N2—C8—C9	115.24 (11)	C26—C27—C28	120.1 (2)
N1—C8—C9	110.82 (10)	C26—C27—H27	120.0
N2—C8—H8	110.0	C28—C27—H27	120.0
N1—C8—H8	110.0	C25—C26—C27	120.27 (18)
C9—C8—H8	110.0	C25—C26—H26	119.9
C10—C9—C14	119.01 (13)	C27—C26—H26	119.9
C10—C9—C8	120.87 (12)	C26—C25—C24	120.25 (18)
C14—C9—C8	120.04 (12)	C26—C25—H25	119.9
C11—C10—C9	120.82 (13)	C24—C25—H25	119.9
C11—C10—H10	119.6	C23—C24—C25	119.98 (18)
C9—C10—H10	119.6	C23—C24—H24	120.0
C12—C11—C10	119.08 (14)	C25—C24—H24	120.0
C12—C11—H11	120.5	C22—N1—C8	113.53 (11)
C10—C11—H11	120.5	C22—N1—S1	123.54 (9)
C13—C12—C11	121.32 (14)	C8—N1—S1	122.84 (9)
C13—C12—Cl1	120.00 (12)	C15—N2—C21	122.66 (12)
C11—C12—Cl1	118.67 (12)	C15—N2—C8	121.51 (11)
C12—C13—C14	119.14 (14)	C21—N2—C8	113.92 (11)
C12—C13—H13	120.4	O3—C22—N1	126.55 (13)
C14—C13—H13	120.4	O3—C22—C21	126.26 (13)
C9—C14—C13	120.49 (14)	N1—C22—C21	107.17 (11)
C9—C14—H14	119.8	O1—S1—O2	120.96 (7)
C13—C14—H14	119.8	O1—S1—N1	106.58 (6)
C16—C15—N2	120.96 (13)	O2—S1—N1	104.10 (6)
C16—C15—C20	118.45 (14)	O1—S1—C5	108.92 (7)
N2—C15—C20	120.58 (14)	O2—S1—C5	109.35 (7)
C17—C16—C15	120.01 (15)	N1—S1—C5	105.82 (6)
C17—C16—H16	120.0		
			
C7—C2—C3—C4	−0.3 (3)	C27—C26—C25—C24	−0.8 (3)
C1—C2—C3—C4	179.17 (17)	C28—C23—C24—C25	0.1 (3)
C2—C3—C4—C5	0.7 (3)	C21—C23—C24—C25	−174.97 (15)
C3—C4—C5—C6	−0.5 (2)	C26—C25—C24—C23	0.4 (3)
C3—C4—C5—S1	176.25 (13)	N2—C8—N1—C22	−14.57 (14)
C4—C5—C6—C7	−0.1 (2)	C9—C8—N1—C22	107.61 (13)
S1—C5—C6—C7	−176.75 (13)	N2—C8—N1—S1	168.83 (9)
C3—C2—C7—C6	−0.2 (3)	C9—C8—N1—S1	−69.00 (14)
C1—C2—C7—C6	−179.74 (17)	C16—C15—N2—C21	177.49 (13)
C5—C6—C7—C2	0.4 (3)	C20—C15—N2—C21	−3.5 (2)
N2—C8—C9—C10	45.41 (17)	C16—C15—N2—C8	14.2 (2)
N1—C8—C9—C10	−67.56 (15)	C20—C15—N2—C8	−166.84 (13)
N2—C8—C9—C14	−137.83 (13)	C22—C21—N2—C15	−170.26 (12)
N1—C8—C9—C14	109.21 (14)	C23—C21—N2—C15	71.64 (17)
C14—C9—C10—C11	−2.5 (2)	C22—C21—N2—C8	−5.78 (16)
C8—C9—C10—C11	174.27 (13)	C23—C21—N2—C8	−123.89 (13)
C9—C10—C11—C12	−0.5 (2)	N1—C8—N2—C15	176.67 (12)
C10—C11—C12—C13	3.2 (2)	C9—C8—N2—C15	57.67 (17)
C10—C11—C12—Cl1	−175.62 (11)	N1—C8—N2—C21	11.99 (15)
C11—C12—C13—C14	−2.8 (2)	C9—C8—N2—C21	−107.01 (14)
Cl1—C12—C13—C14	176.03 (12)	C8—N1—C22—O3	−169.50 (14)
C10—C9—C14—C13	2.9 (2)	S1—N1—C22—O3	7.1 (2)
C8—C9—C14—C13	−173.88 (13)	C8—N1—C22—C21	11.90 (15)
C12—C13—C14—C9	−0.3 (2)	S1—N1—C22—C21	−171.52 (9)
N2—C15—C16—C17	179.67 (14)	N2—C21—C22—O3	177.75 (14)
C20—C15—C16—C17	0.7 (2)	C23—C21—C22—O3	−59.49 (19)
C15—C16—C17—C18	0.6 (2)	N2—C21—C22—N1	−3.64 (15)
C16—C17—C18—C19	−1.1 (3)	C23—C21—C22—N1	119.12 (13)
C17—C18—C19—C20	0.4 (3)	C22—N1—S1—O1	38.67 (13)
C18—C19—C20—C15	0.9 (3)	C8—N1—S1—O1	−145.07 (10)
C16—C15—C20—C19	−1.4 (2)	C22—N1—S1—O2	167.58 (11)
N2—C15—C20—C19	179.58 (14)	C8—N1—S1—O2	−16.15 (12)
N2—C21—C23—C24	−145.95 (14)	C22—N1—S1—C5	−77.18 (12)
C22—C21—C23—C24	99.02 (16)	C8—N1—S1—C5	99.08 (11)
N2—C21—C23—C28	39.0 (2)	C6—C5—S1—O1	149.38 (12)
C22—C21—C23—C28	−76.01 (18)	C4—C5—S1—O1	−27.35 (14)
C24—C23—C28—C27	−0.2 (3)	C6—C5—S1—O2	15.21 (14)
C21—C23—C28—C27	174.86 (17)	C4—C5—S1—O2	−161.53 (12)
C23—C28—C27—C26	−0.2 (3)	C6—C5—S1—N1	−96.37 (13)
C28—C27—C26—C25	0.7 (4)	C4—C5—S1—N1	86.89 (13)

### Hydrogen-bond geometry (Å, °)

**Table:**

Cg2 and Cg4 are the centroids of the C2–C7 and C15–C20 rings, respectively.

**Table:**

*D*—H···*A*	*D*—H	H···*A*	*D*···*A*	*D*—H···*A*
C6—H6···O2	0.93	2.59	2.934 (2)	102
C8—H8···O3^i^	0.98	2.44	3.3659 (17)	158
C14—H14···O3^i^	0.93	2.57	3.3855 (19)	146
C24—H24···O2^ii^	0.93	2.59	3.289 (2)	132
C1—H1C···Cg4^iii^	0.96	2.90	3.484 (2)	120
C11—H11···Cg2^iii^	0.93	2.88	3.619 (17)	138

Symmetry codes:  (i) −*x*+3/2, *y*−1/2, −*z*+1/2; (ii) −*x*+3/2, *y*+1/2, −*z*+1/2; (iii) −*x*+1, −*y*+1, −*z*.
